# Trastuzumab Emtansine–Associated Porto-Sinusoidal Vascular Disorder: Clinical Features and Outcomes from Published Cases

**DOI:** 10.3390/jcm15134950

**Published:** 2026-06-25

**Authors:** Jiazheng Sun, Yanjie Lin, Hong Zhao

**Affiliations:** Department of Infectious Disease, Center for Liver Disease, Peking University First Hospital, Beijing 100034, China; 2411210205@stu.pku.edu.cn (J.S.); 2311110264@bjmu.edu.cn (Y.L.)

**Keywords:** ado-trastuzumab emtansine, porto-sinusoidal vascular disorder, portal hypertension

## Abstract

**Introduction**: Ado-trastuzumab emtansine (T-DM1) is a targeted agent for human epidermal growth factor receptor 2 (HER2)-positive breast cancer, which combines the anti-tumor activity of trastuzumab with the cytotoxic effect of DM1, a microtubule inhibitor. Although T-DM1 has improved outcomes in patients with HER2-positive breast cancer, portal hypertension may occur during treatment in the absence of overt cirrhosis on liver biopsy. These clinical and pathological features are consistent with porto-sinusoidal vascular disorder (PSVD). This study aimed to summarize the reported clinical, biochemical, imaging, histological, therapeutic, and prognostic features of T-DM1-associated PSVD. **Methods**: PubMed and Web of Science were searched for published cases of T-DM1-associated PSVD. Given the evolving terminology of PSVD, related terms, including non-cirrhotic portal hypertension and nodular regenerative hyperplasia, were also included in the search strategy. If the patient has a recorded history of T-DM1 exposure and the liver biopsy results meet PSVD criteria, the case is included regardless of whether there is clinical, endoscopic, or imaging evidence of portal hypertension. Cases without liver biopsy or with features suggestive of overt cirrhosis were excluded. Patient-level data were extracted and descriptively summarized, including demographic characteristics, clinical manifestations, biochemical indicators, imaging examination results, liver biopsy results, treatment methods, and prognosis. Unreported data were considered missing values and were not imputed. **Results**: Seven eligible articles comprising eight patients were identified. All patients were female, with a mean age of 60.38 years and a median age of 62.50 years. The interval from T-DM1 initiation to PSVD diagnosis ranged from 6 to 30 months. When reported, the mean interval from treatment initiation to symptom onset was 18.3 months. Thrombocytopenia and splenomegaly were observed in 7 of 8 patients. Mild elevations in alanine aminotransferase and aspartate aminotransferase were observed in all patients. Liver biopsy showed thinned and disorganized hepatic plates accompanied by nodular regeneration of hepatocytes in six patients. Clinical improvement was observed after discontinuation or modification of T-DM1 in most cases. **Conclusions**: T-DM1-associated PSVD is a rare but clinically significant complication that may develop months after treatment initiation. It commonly presents with thrombocytopenia, splenomegaly, gastrointestinal bleeding, or mild liver biochemical abnormalities in the absence of overt cirrhosis. Early recognition of unexplained platelet decline, splenic enlargement, or portal hypertension-related findings during T-DM1 therapy may facilitate timely diagnosis and individualized management. Withdrawal or modification of the suspected drug may contribute to clinical improvement, although further studies are needed to clarify the mechanism and optimal management strategy.

## 1. Introduction

Porto-sinusoidal vascular disorder (PSVD) is a hepatic vascular disorder characterized by histological lesions, involving the small intrahepatic portal veins or hepatic sinusoids in the absence of obvious cirrhosis. It may present with or without clinical manifestations of portal hypertension, and the diagnosis is established on the basis of liver biopsy, with key pathological features including obliterative portal venopathy, nodular regenerative hyperplasia, and incomplete septal cirrhosis [1,2]. In patients with portal hypertension, hepatic functional reserve is generally well preserved, with mild elevation or near-normal levels of hepatic and biliary enzymes [3].

Ado-trastuzumab emtansine (T-DM1) is an antibody–drug conjugate used for HER2-positive breast cancer. It combines the HER2-targeting activity of trastuzumab with the cytotoxic activity of DM1, a microtubule inhibitor, thereby enabling targeted delivery of chemotherapy to HER2-expressing tumor cells [4,5]. T-DM1 has demonstrated clinically meaningful survival benefits in patients with HER2-positive advanced breast cancer. However, treatment-related adverse events, particularly thrombocytopenia and hepatotoxicity, deserve clinical attention [5].

The liver and reticuloendothelial system are major sites of T-DM1 catabolism, and elimination occurs predominantly through fecal and biliary routes. Approximately 80% of eliminated T-DM1 is excreted in feces and 50% in bile. This pharmacokinetic profile may partly explain the susceptibility to hepatobiliary adverse events during T-DM1 therapy [6]. With the increasing clinical use of T-DM1, reports of drug-related hepatic injury have gradually accumulated. Among these complications, T-DM1-associated porto-sinusoidal vascular disorder (PSVD) is rare but clinically non-negligible because it may present with portal hypertension, thrombocytopenia, splenomegaly, or mild liver biochemical abnormalities in the absence of cirrhosis, which are easily overlooked in clinical practice.

The first well-documented case of T-DM1-associated PSVD was reported as idiopathic non-cirrhotic portal hypertension, a condition that is now more appropriately classified within the current concept of PSVD [2]. Because the reported cases remain limited and the terminology used in previous reports has been heterogeneous, this literature-based pooled case analysis aimed to summarize the clinical features, biochemical abnormalities, imaging findings, histological characteristics, management, and outcomes of T-DM1-associated PSVD.

## 2. Methods

### 2.1. Study Design

This study was designed as a literature-based pooled case analysis of published reports describing trastuzumab emtansine (T-DM1)-associated porto-sinusoidal vascular disorder (PSVD). Because only a limited number of cases have been reported and individual studies were mainly case reports or small case series, the available evidence was summarized descriptively at the patient level.

### 2.2. Literature Search

A literature search was conducted in PubMed and Web of Science to identify published cases of T-DM1-associated PSVD. The search was performed from database inception to 30 July 2025. Search terms included combinations of terms related to the drug exposure and hepatic vascular disorder, including “trastuzumab emtansine,” “T-DM1,” “ado-trastuzumab emtansine,” “porto-sinusoidal vascular disorder,” “PSVD,” “non-cirrhotic portal hypertension,” “idiopathic non-cirrhotic portal hypertension,” “nodular regenerative hyperplasia,” and “obliterative portal venopathy.” Because the terminology of PSVD has evolved over time, earlier diagnostic terms such as non-cirrhotic portal hypertension and nodular regenerative hyperplasia were also considered. The reference lists of relevant articles were additionally reviewed to identify potentially eligible reports.

### 2.3. Eligibility Criteria

Published cases were considered eligible if they met the following criteria:

(1) Documented exposure to T-DM1;
(2) Clinical, endoscopic, radiological, or laboratory findings suggestive of portal hypertension or porto-sinusoidal vascular injury;
(3) Liver biopsy findings compatible with PSVD, including nodular regenerative hyperplasia, obliterative portal venopathy, incomplete septal fibrosis, or other non-cirrhotic vascular-pattern liver lesions.

Cases were excluded if liver biopsy was not available, if cirrhosis was reported histologically or radiologically, or if portal hypertension was more plausibly explained by another established cause, such as advanced cirrhosis, diffuse hepatic metastatic infiltration, or another clearly documented chronic liver disease. When multiple publications appeared to describe the same patient, the most complete report was used for data extraction.

### 2.4. Data Extraction

Patient-level data were extracted from each eligible report. The extracted variables included demographic characteristics, underlying malignancy, previous anticancer treatments, duration of T-DM1 exposure, interval from T-DM1 initiation to symptom onset or diagnosis, presenting manifestations, platelet count, liver biochemical parameters, coagulation function, imaging findings, endoscopic findings, liver biopsy findings, management strategies, and clinical outcomes. Particular attention was paid to features related to portal hypertension, including thrombocytopenia, splenomegaly, varices, gastrointestinal bleeding, ascites, and radiological evidence of portal venous abnormalities.

### 2.5. Data Synthesis and Statistical Analysis

The final set of eight cases was identified after screening titles, abstracts, full texts, and reference lists of relevant articles. Given the small number of published cases and the heterogeneity of case reporting, all data were summarized descriptively. Continuous variables were reported as mean, median, and range when available. Categorical variables were summarized as numbers and percentages. Missing or unreported variables were treated as unavailable and were not imputed.

## 3. Results and Discussion

### 3.1. Case Identification and Overview of Included Patients

Seven eligible articles were identified, comprising a total of eight patients with T-DM1-associated porto-sinusoidal vascular disorder. The main clinical, biochemical, imaging, histological, therapeutic, and prognostic features of the included cases are summarized in Table 1. All reported patients were female, with a mean age of 60.38 years and a median age of 62.50 years. PSVD was diagnosed 6–30 months after the initiation of T-DM1 therapy, with a mean interval of 18.3 months from treatment initiation to symptom onset [7,8,9,10,11,12,13].

### 3.2. Clinical Presentation and Subclinical Clues

The clinical presentation of T-DM1-associated PSVD was mainly characterized by manifestations of portal hypertension in the setting of preserved or only mildly impaired liver function. Gastrointestinal bleeding and thrombocytopenia were common initial manifestations. During the disease course, thrombocytopenia and splenomegaly were observed in 7 of 8 patients. Mild elevations in alanine aminotransferase and aspartate aminotransferase were reported in all patients, whereas severe hepatic dysfunction was not a dominant feature.

These findings suggest that T-DM1-associated PSVD may initially present with subtle or non-specific abnormalities rather than distinct hepatic failure. In particular, progressive thrombocytopenia, splenomegaly, mild liver enzyme elevation, or imaging evidence of portal hypertension during T-DM1 therapy may represent early clinical or subclinical clues. Therefore, PSVD should be considered when these abnormalities occur in patients receiving T-DM1, even in the absence of obvious cirrhosis or marked impairment of hepatic synthetic function.

### 3.3. Biochemical, Imaging, and Histological Findings

The biochemical profile of the included cases was generally characterized by mild liver enzyme abnormalities, while liver function was relatively preserved. This pattern is consistent with the clinical nature of PSVD, in which portal hypertension may develop without the typical biochemical features of advanced cirrhosis.

Imaging findings were mainly related to portal hypertension, particularly splenomegaly and secondary vascular changes. However, imaging alone was insufficient to establish the diagnosis. Liver biopsy played a central role in case identification, especially because cases with overt cirrhosis were excluded. Histologically, six patients showed thinned and disorganized hepatic plates accompanied by nodular regeneration of hepatocytes [9,10,11,12,13]. The pathological findings were consistent with nodular regenerative hyperplasia (NRH), with prominent proliferative hepatocellular nodules compressing the intrahepatic portal vein branches visible under light microscopy. Intrahepatic portal vein occlusion was reported in two cases, accompanied by incomplete periportal septal fibrosis: fibrosis formed in the periportal regions but presented as an incomplete septal pattern (non-continuous fibrous septa), with no typical cirrhotic pseudolobule formation. In some cases, lymphocytic infiltration in the portal tracts and interface hepatitis were also described, suggesting overlapping features of drug-induced liver injury. However, the presence of nodular regenerative hyperplasia, portal venopathy, and incomplete septal fibrosis supported a vascular-pattern liver injury consistent with PSVD rather than conventional cirrhosis.

### 3.4. Management, Outcomes, and Clinical Implications

Based on the clinical reports summarized in this study, the monitoring of platelet count, liver biochemical parameters, and abdominal imaging findings may help identify early features of T-DM1-associated PSVD. Before T-DM1 treatment, baseline assessment of liver biochemical parameters, complete blood count, coagulation function, and abdominal imaging may be useful, especially in patients with previous exposure to chemotherapeutic agents or other risk factors for hepatic vascular injury.

During treatment, progressive thrombocytopenia, newly developed splenomegaly, persistent mild liver enzyme abnormalities, or radiological signs of portal hypertension may warrant further evaluation for PSVD. In patients with mild laboratory abnormalities but no evidence of portal hypertension, close monitoring may be appropriate. In patients with worsening thrombocytopenia, hyperbilirubinemia, splenomegaly, or suspected varices, further assessment for portal hypertension should be taken into account. When severe portal hypertension-related complications occur, discontinuation of T-DM1 and individualized management in collaboration with oncology and hepatology specialists may be required.

Symptom resolution or clinical improvement was reported in most evaluable cases after discontinuation or modification of T-DM1. This finding suggests that timely recognition of T-DM1-associated PSVD and withdrawal of the suspected agent may be important for improving clinical outcomes. With the exception of one patient who died from progressive hepatic metastases, all other patients showed varying degrees of improvement in liver function and platelet counts within one year after discontinuation or modification of T-DM1. Serial radiological follow-up did not reveal progressive portal hypertension or newly developed clinical complications in these cases. Compared with cirrhosis-related portal hypertension, PSVD is generally characterized by relatively preserved liver function and a lower incidence of severe hepatic failure-related complications, such as jaundice, ascites, and liver failure [14]. However, because of its insidious course, patients may already have portal hypertension-related manifestations, including high-risk esophagogastric varices, at the time of diagnosis. In the largest multicenter cohort of 587 patients with PSVD and portal hypertension, 36% had portal hypertension-related complications at diagnosis. During follow-up, the 5-year cumulative incidence of first bleeding was 15% among patients without bleeding at presentation, the 5-year rebleeding rate was 18%, and the 5-year cumulative incidences of new or worsening ascites and portal vein thrombosis were 18% and 16%, respectively. Liver transplantation was required in 8.5% of patients, and transplant-free survival was 97% at 1 year and 83% at 5 years [13].

Considering the relatively limited case reports available, these observations are still inadequate to be summarized as a standardized management algorithm. Instead, the available cases indicate that clinicians should maintain a high index of suspicion for PSVD when patients receiving T-DM1 develop thrombocytopenia, splenomegaly, gastrointestinal bleeding, or persistent mild liver biochemical abnormalities.

### 3.5. Possible Mechanism of T-DM1-Associated PSVD

The underlying mechanism by which T-DM1 induces PSVD remains unclear. T-DM1 combines the HER2-targeting activity of trastuzumab with the cytotoxic activity of DM1, a microtubule inhibitor. DM1 binds to tubulin, inhibits microtubule polymerization, and causes cell cycle arrest at the metaphase, the formation of multinucleated cells and abnormal mitotic figures, ultimately leading to mitotic catastrophe [15]. Furthermore, T-DM1 has been demonstrated to retain the mechanistic actions of trastuzumab, including the blockade of the HER2 signaling pathway, inhibition of HER2 extracellular domain shedding, and activation of the innate and adaptive anti-tumor immune responses [16]. Previous studies have suggested that chemotherapeutic agents may induce injury in the hepatic sinusoidal region, leading to sinusoidal wall damage, hepatic stellate cell activation, and stromal deposition [17].

At present, direct HER2-mediated toxicity to hepatic sinusoidal endothelial cells has not been clearly established. Nevertheless, pathological findings in reported cases suggest injury and obstruction at the sinusoidal or porto-sinusoidal level. Experimental evidence has also suggested that T-DM1 may promote hepatic inflammatory responses. Yan et al. [18] reported in a mouse model that T-DM1 increased hepatic TNF-α expression and promoted neutrophil accumulation in the liver. Therefore, T-DM1-associated PSVD may involve indirect hepatocellular or sinusoidal injury, followed by vascular obstruction, remodeling, and nodular regenerative changes. Further cellular and tissue-level studies are required to clarify the pathogenic mechanisms underlying T-DM1-associated PSVD.

### 3.6. Limitations

This study has several limitations. First, the analysis was based on a small number of published cases, which limit the generalizability of the findings. Second, the included reports were mainly case reports or small case series, with heterogeneous and incomplete reporting of clinical, biochemical, imaging, histological, and follow-up data. Third, publication bias is possible, because severe or unusual cases are more likely to be reported. Fourth, owing to the descriptive nature of the available evidence, a definitive causal relationship between T-DM1 exposure and PSVD cannot be established. Finally, the evolving terminology of PSVD may have led to underrecognition or inconsistent classification in earlier reports.

## 4. Conclusions

T-DM1-associated PSVD is a rare but clinically important hepatic vascular complication that may occur months after treatment initiation. Reported cases were commonly characterized by thrombocytopenia, splenomegaly, gastrointestinal bleeding, and normal or mildly abnormal liver biochemical parameters in the absence of cirrhosis. Liver biopsy remains important for distinguishing PSVD from cirrhosis-related portal hypertension.

Although hepatic synthetic function is often preserved in PSVD, the clinical course is not always benign once portal hypertension develops. Early recognition of unexplained platelet decline, splenic enlargement, mild liver enzyme abnormalities, or radiological signs of portal hypertension during T-DM1 therapy may facilitate timely diagnosis and individualized management. Discontinuation or modification of T-DM1 may contribute to clinical improvement, although further studies are required to clarify the underlying mechanisms, risk factors, and optimal management strategies.

## Figures and Tables

**Table 1:** Summary of reported cases of T-DM1-associated porto-sinusoidal vascular disorder *.

Case	Reference	Age/Sex	Primary Malignancy/Receptor Status	T-DM1 Exposure Before Diagnosis	Clinical Presentation/Diagnostic Clue	Laboratory/Platelet Findings	Imaging Findings	Key Histological Findings	PSVD Management	Outcome
1	Antunes et al. [7], 2025	66/F	ER-, PR-, HER2+ invasive ductal carcinoma	T-DM1 every 3 weeks with pertuzumab for 15 months	Abdominal tenderness and ascites with spontaneous bacterial peritonitis	ALT/AST and GGT/ALP normal; thrombocytopenia: NR	Patent hepatic vessels; HVPG 13 mmHg; portal vein thrombosis treated	Centrilobular sinusoidal dilatation/congestion; nodular hepatic parenchyma; no inflammation or fibrosis; periportal sinusoidal CD34 staining	T-DM1 and pertuzumab discontinued; enoxaparin for portal vein thrombosis	Biochemical parameters and platelet count improved; ascites resolved on follow-up CT.
2	Antunes et al. [7], 2025	50/F	HER2+, ER-, PR- ductal carcinoma in situ of the left breast	T-DM1 every 3 weeks with pertuzumab for 15 months	Transaminase elevation, thrombocytopenia, and CT signs of portal hypertension	ALT elevated to 2*ULN; thrombocytopenia: Yes	CT signs of portal hypertension without metastasis; small esophageal varices	Alternating hepatic plate atrophy/regeneration; patchy CD34 staining outside portal areas; mild macrovesicular steatosis; ballooned hepatocytes and Mallory bodies	T-DM1 discontinued	Liver function improved within 1 month; thrombocytopenia resolved; no ascites or hepatic decompensation
3	Fernandes et al. [8], 2025	61/F	Invasive ductal carcinoma, pT1cN0M0, ER+, HER2+	T-DM1 3.6 mg/kg; 27 months	CT showed inferior mesenteric venous varices and portal hypertension; no significant symptoms such as ascites	AST and ALT >2*ULN; thrombocytopenia: Yes	Small ascites; no portal vein thrombosis or hepatic metastasis	Sinusoidal dilatation/congestion with collagen fiber proliferation; interface hepatitis; no cirrhosis; hepatic plate disarray	T-DM1 discontinued	PSVD-related laboratory abnormalities improved; patient later died due to cancer progression
4	Force et al. [9], 2016	52/F	Lung adenocarcinoma, HER2+, PD-L1 high expression	T-DM1 3.6 mg/kg every 3 weeks; 18 months	New-onset cholestasis, elevated bilirubin, and progressive platelet decrease	Elevated GGT, ALP, and total bilirubin; thrombocytopenia: Yes	Portal vein thrombosis and splenomegaly; no hepatic metastasis; no varices; liver stiffness 5.5 kPa	Diffuse nodules of proliferative hepatocytes compressing adjacent plates with sinusoidal dilatation	T-DM1 dose reduced to 1.8 mg/kg for 3 weeks	Reduced-dose T-DM1 continued for >9 months without portal hypertension or cancer progression
5	Garrido et al. [10], 2022	48/F	Invasive ductal carcinoma with high HER2 overexpression	T-DM1 3.6 mg/kg; 12 months	Hepatosplenomegaly with mild liver function abnormalities	Mild transaminase elevation; thrombocytopenia: No	Paraumbilical vein recanalization and splenomegaly; no ascites or cirrhosis; hepatic metastases later confirmed	NRH-like sinusoidal lesions; nodular and atrophic areas; no cirrhosis or portal tract fibrosis; thickened hepatic plates in hyperplastic nodules	T-DM1 discontinued; subsequent trastuzumab monotherapy and chemotherapy regimens	Liver function normalized 4 months later; oncologic disease progressed rapidly
6	Hassan et al. [11], 2020	73/F	ER+, PR-, HER2+, T3N2 left breast cancer	T-DM1 3.6 mg/kg; 15 months	Chronic liver disease with nodularity, splenomegaly, and esophageal varices	AST/ALT/ALP fluctuated but stayed <2.5x ULN; thrombocytopenia: Yes	Nodular liver morphology; ligamentum teres patency; fissure and left-segment enlargement; esophageal varices	Nodule-like expansion of liver cell cords; NRH supported by reticulin stain; biliary duct injury; CK7 findings indicating biliary metaplasia	T-DM1 discontinued	AST/ALT normalized 6 months later; ALP improved but remained <1.5x ULN; platelet count improved but did not normalize
7	Prochaska et al. [12], 2016	43/F	Grade 3 invasive breast carcinoma, HER2+++, hormone receptor-negative, initially cT2N+ cM0	T-DM1 dose reduction sequence: 3.6, 3.0, then 2.4 mg/kg	CT 18 months after T-DM1 showed portal vein enlargement and new splenomegaly	Grade 1 transaminase and GGT elevation; hyperbilirubinemia increased to grade 2; thrombocytopenia: Yes	Portal vein enlargement and newly detected splenomegaly	Preserved trabecular architecture; dilated vessels in periphery of portal spaces; mild sinusoidal enlargement	T-DM1 discontinued; spironolactone and torasemide administered	Tumor stable; no progression of PSVD-related symptoms and no severe complications
8	Lepelley et al. [13], 2018	69/F	Right-sided HER2-positive invasive breast cancer	T-DM1 3.6 mg/kg; 10 months	ALT and AST increased to 3*ULN with thrombocytopenia	Aminotransferases (ALT/AST) increased to 3x normal; thrombocytopenia: Yes	Small amount of ascites; no splenomegaly, portal vein thrombosis, or vascular variations	Portal vessel occlusion; sinusoidal obstruction with lobular congestion; incomplete septal fibrosis; no pseudolobules or cirrhosis	T-DM1 discontinued; furosemide plus spironolactone; lactulose for encephalopathy	At 1-year follow-up, symptoms relieved, liver function stable, and no complications observed

* Data were extracted from seven published articles comprising eight reported patients. Abbreviations: ALP, alkaline phosphatase; ALT, alanine aminotransferase; AST, aspartate aminotransferase; CT, computed tomography; ER, estrogen receptor; GGT, gamma-glutamyl transferase; HER2, human epidermal growth factor receptor 2; HVPG, hepatic venous pressure gradient; NR, not reported; NRH, nodular regenerative hyperplasia; PD-L1, programmed death-ligand 1; PR, progesterone receptor; PSVD, porto-sinusoidal vascular disorder; T-DM1, ado-trastuzumab emtansine; ULN, upper limit of normal; NR, not reported.

## Data Availability

All data analyzed in this study were extracted from previously published articles cited in this manuscript. No new datasets were generated.

## Abbreviations

Abbreviation	Full name
PSVD	porto-sinusoidal vascular disorder
T-DM1	Ado-trastuzumab emtansine
HER2	epidermal growth factor receptor 2
INCPH	Idiopathic Non-cirrhotic Portal Hypertension
BC	breast cancer
ER	Estrogen Receptor
PR	Progesterone Receptor
ALT	Alanine Aminotransferase
GGT	Gamma-Glutamyl Transferase
AST	Aspartate Aminotransferase
ALP	Alkaline Phosphatase
ULN	Upper Limit of Normal
HVPG	Hepatic venous pressure gradient
NRH	Nodular Regenerative Hyperplasia
CT	Computed tomography
MRI	Magnetic Resonance Imaging
AFP	Alpha-fetoprotein
